# Progression of Cartilage Degradation, Bone Resorption and Pain in Rat Temporomandibular Joint Osteoarthritis Induced by Injection of Iodoacetate

**DOI:** 10.1371/journal.pone.0045036

**Published:** 2012-09-11

**Authors:** Xue-Dong Wang, Xiao-Xing Kou, Dan-Qing He, Min-Min Zeng, Zhen Meng, Rui-Yun Bi, Yan Liu, Jie-Ni Zhang, Ye-Hua Gan, Yan-Heng Zhou

**Affiliations:** 1 Department of Orthodontics, Peking University School and Hospital of Stomatology, Beijing, China; 2 Center for Temporomandibular Disorders and Orofacial Pain, Peking University School and Hospital of Stomatology, Beijing, China; University of Massachusetts Medical, United States of America

## Abstract

**Background:**

Osteoarthritis (OA) is an important subtype of temporomandibular disorders. A simple and reproducible animal model that mimics the histopathologic changes, both in the cartilage and subchondral bone, and clinical symptoms of temporomandibular joint osteoarthritis (TMJOA) would help in our understanding of its process and underlying mechanism.

**Objective:**

To explore whether injection of monosodium iodoacetate (MIA) into the upper compartment of rat TMJ could induce OA-like lesions.

**Methods:**

Female rats were injected with varied doses of MIA into the upper compartment and observed for up to 12 weeks. Histologic, radiographic, behavioral, and molecular changes in the TMJ were evaluated by light and electron microscopy, MicroCT scanning, head withdrawal threshold test, real-time PCR, immunohistochemistry, and TUNEL assay.

**Results:**

The intermediate zone of the disc loosened by 1 day post-MIA injection and thinned thereafter. Injection of an MIA dose of 0.5 mg or higher induced typical OA-like lesions in the TMJ within 4 weeks. Condylar destruction presented in a time-dependent manner, including chondrocyte apoptosis in the early stages, subsequent cartilage matrix disorganization and subchondral bone erosion, fibrosis, subchondral bone sclerosis, and osteophyte formation in the late stages. Nociceptive responses increased in the early stages, corresponding to severe synovitis. Furthermore, chondrocyte apoptosis and an imbalance between anabolism and catabolism of cartilage and subchondral bone might account for the condylar destruction.

**Conclusions:**

Multi-level data demonstrated a reliable and convenient rat model of TMJOA could be induced by MIA injection into the upper compartment. The model might facilitate TMJOA related researches.

## Introduction

Temporomandibular joint osteoarthritis (TMJOA) is an important subtype of temporomandibular disorders (TMD) [1], [2] and is especially common in female patients with severe pain and dysfunction of the temporomandibular joint (TMJ) [3], [4]. OA is characterized by a progressive degradation of cartilage, subchondral bone remodeling, synovitis, and chronic pain [3], [5], [6].

However, the process of TMJOA remains obscure. Chondrocyte death due to either apoptosis or necrosis is assumed to be a central feature in the degeneration of osteoarthritic cartilage and to contribute to the development of clinical or experimental OA [7], [8]. Resorption and abrasion of condylar subchondral bone are unique in TMJOA, which usually shows no typical pannus in the synovium whereas rheumatoid arthritis does [3]. Therefore, a proper animal model may provide a useful way to understand the pathogenesis of TMJOA and to evaluate potential therapeutic interventions [9].

Thus far, several methods have attempted to create animal models of TMJOA, including surgical [10], mechanical [11], drug-inducing [12], [13], and spontaneously occurring methods [14]. Due to the limited availability of special animal species, slow progression of the disease, and complicated operations, the use of spontaneous or surgical-induced methods was limited [13], [15]. In addition, the lack of progressive changes led to a number of drug-induced models being merely models of cartilage damage rather than OA [9]. A simple and reproducible animal model of TMJOA that mimics the histopathologic changes both in cartilage and subchondral bone, as well as clinical symptoms, is still needed.

Intra-articular injection of monosodium iodoacetate (MIA) to induce OA-like lesions is widely used to induce knee OA [16], [17], [18], [19], [20], [21]. MIA mainly inhibits the activity of glyceraldehyde-3-phosphate dehydrogenase leading to apoptosis of chondrocytes [18], [20], [22]. The MIA-induced OA model has the great advantage of easy modulation of the progression and severity of the articular lesions by modification of MIA concentration [19]. Although a few studies have attempted to induce OA-like lesions in rabbit TMJ by MIA injection into the lower compartment with or without surgical assistance [13], [23], [24], it is important to explore whether MIA could induce OA-like lesions in the rat TMJ, since rats are one of the most widely used species in experimental research and drug toxicology testing [15]. The TMJ is partitioned by a disc, which forms a larger upper and smaller lower compartment. Agent injection into the lower compartment is a difficult procedure both in humans and animals because of its limited space [25], whereas injection into the upper compartment, even in rats, is technically and manually preferable, and has often been used previously [26], [27], [28].

The question then arises as to whether MIA injection into the upper compartment of the rat TMJ can be used to create a comprehensive OA model. To address this question, investigations at the histopathologic, radiographic, molecular, and noceiceptive behavioral levels were performed in this study to examine whether injection of MIA into the upper compartment of the rat TMJ could induce OA-like lesions in the entire joint.

## Materials and Methods

### Ethics Statement

With the approval of the Peking University Institutional Animal Care and Use Committee (NO: LA2012-59), all rats were housed under controlled temperatures in a 12 h light/dark cycle with easy access to food and water.

### Induction of TMJOA

**Figure 1:**
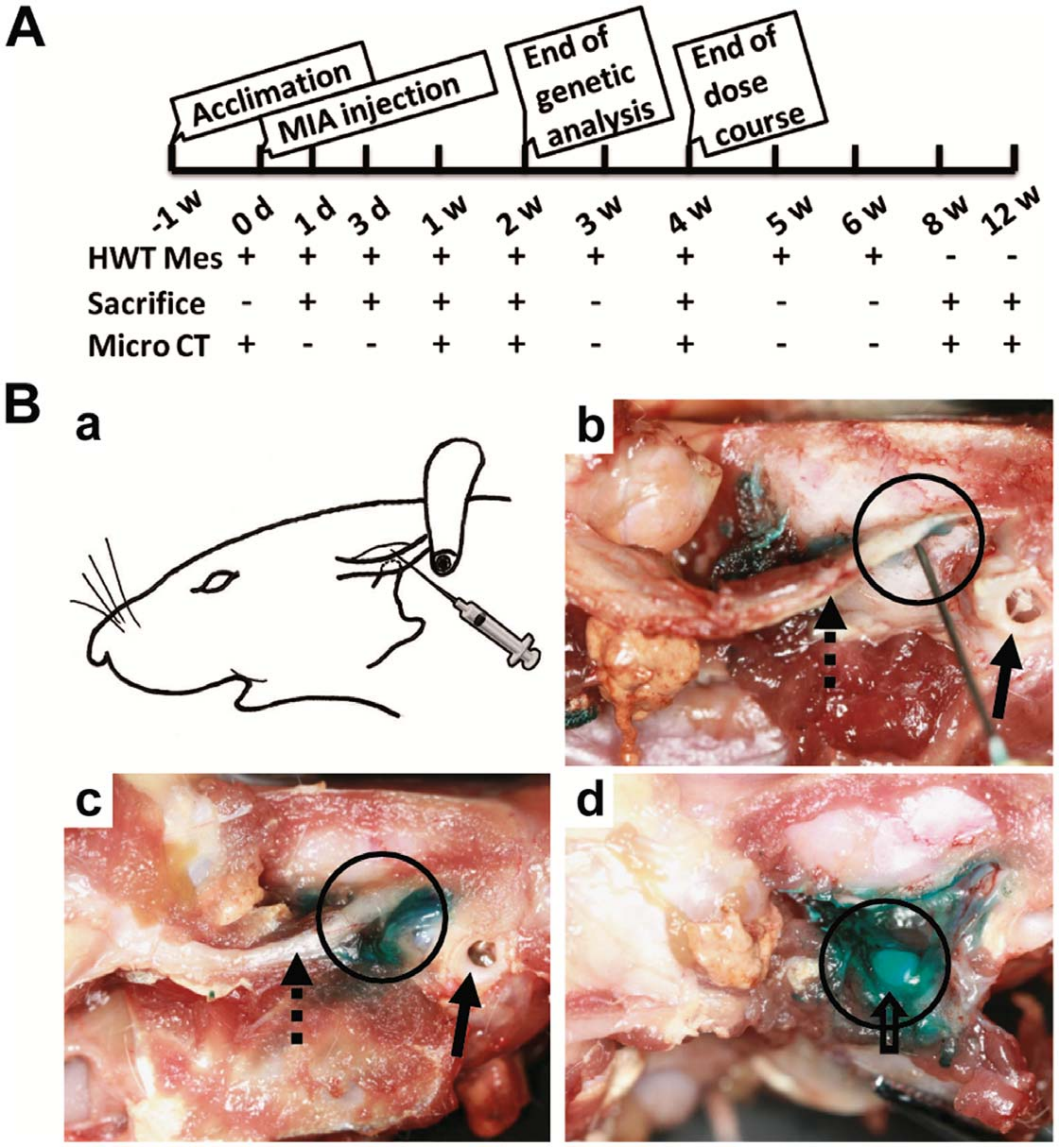
Outline of experimental design and confirmation of injection site into upper compartment of rat TMJ. A: Outline of experimental design. B: Photograph of dye (fast green solution) injection into the upper compartment of the left TMJ. (a). Needle insertion was 5 mm anterior to the external auditory canal. (b). Dissection showed that the needle was right under the root of the zygoma (dotted arrow), anterior of the external auditory canal (arrow), stopped at the temporal fossa, and was located in the upper compartment (black circle). (c). 50 µL dye was injected. (d). Opening the capsule revealed that the dye was restricted to the upper compartment of the TMJ (disc and condyle: hollow arrow).

A total of 72 female Sprague-Dawley rats (180–200 g) were randomly assigned to either the experimental (n = 42) or control (n = 30) groups. The experimental schedule is illustrated in Fig. 1A. TMJOA was induced by injection of MIA (Sigma, Saint Louis, USA) dissolved in 50 µL saline into the upper compartment of bilateral TMJs using a 27-gauge 0.5-inch needle without surgical assistance. We first confirmed the injection site by injection of 50 µL dye into the upper compartment (Fig. 1B).

### Dose Course

Various doses of MIA (0.05, 0.1, 0.5, 1, or 2 mg) were injected into the upper compartment of bilateral TMJs of rats in five experimental groups (n = 3/group), while 50 µL saline was injected into TMJs of rats in the control group (n = 3). All rats were sacrificed on day 28 post-injection to validate the adequate dose of MIA for further observations (**Fig. 1A**).

### Time Course

After the dose course test, 0.5 mg MIA (minimum effective dose) or saline was injected into TMJs and rats were sacrificed on days 1, 3, 7, 14, 28, 56, or 84 post-injection (n = 3/group) (**Fig. 1A**).

To determine gene expression profiles during the induction of TMJOA, an additional 12 rats, divided into two groups (n = 6/group), were injected with 0.5 mg MIA or saline and sacrificed on day 14 post-injection.

### Head Withdrawal Threshold (HWT) Measurements

The nociceptive behavior of animals was assessed based on the HWT as described previously [27]. HWT measurements were performed pre-MIA/saline injection and on days 1, 3, 7, 14, 21, 28, 35, and 42 post-injection (**Fig. 1A**). The HWT was calculated as a mean value per joint of 3 rats/group.

### Tissue Harvesting

All rats were sacrificed by pentobarbital overdose. For histopathology, the TMJs of two rats in each group were removed bilaterally *en bloc*, fixed in 4% paraformaldehyde, and demineralized in 15% EDTA. For radiographic examination, the bilateral condyles of one rat in each group were dissected.

For real-time PCR analysis, the condyle heads of six rats in each group (0.5 mg MIA for 2 weeks or control) were dissected. Bilateral condyle heads of each rat were pooled for sufficient RNA extraction owing to the difficulty of isolating and acquiring enough cartilage from the small condylar head of rat and the reason that both the cartilage and subchondral bone were affected by MIA.

### Scanning Electron Microscopy (SEM) and Transmission Electron Microscopy (TEM)

TMJ discs and condylar cartilage were dissected from the rats on day 1 post-injection (n = 3/group). SEM and TEM were performed as described previously [29]. Briefly, the samples were fixed with 2.5% fluteraldehyde solution and 1% osmium tetroxide (Sigma). For SEM, the disc section was produced by tearing through the intermediate zone. For TEM, the intermediate zone of the disc or condylar cartilage was embedded in epoxy resin. Ultrathin sections (100 nm) were stained with lead citrate and uranyl acetate.

### Histopathologic Staining

Paraffin-embedded TMJ blocs were sagittally cut in serial sections at a 5-µm thickness. Sections were stained with hematoxylin and eosin (HE) for routine histological evaluation. Safranin O-fast green (S.O) and Toluidine blue (TB) stains were used to evaluate proteoglycans in the cartilage matrix [18].

### MicroCT Examination

Radiographs of condyles were obtained with a high-resolution MicroCT system (Inveon, Siemens, Germany). The specimens were scanned at 60 kV, 300 µA, and 8.5 µm-effective pixel size. The images were analyzed using software provided by the manufacturer. All sagittal images were captured using the same parameters: Ct = −550; W = 550.

### Real-time PCR

Total RNA was isolated from the condylar heads containing cartilage and subchondral bone using TRIzol reagent (Invitrogen, Carlsbad, USA) according to the manufacturer’ s instructions. The condylar heads were ground into powder in liquid nitrogen using a cryogenic grinder (6770 Freezer/Mill, SPEX SamplePrep, NJ, USA). Reverse transcription were performed with an iScript cDNA synthesis kit (Bio-Rad) in 20 µl reaction volume containing 1 µg of total RNA as described previously [27], [30]. Real-time PCR was performed with Power SYBR Green PCR Master Mix (Applied Biosystems) using a 7500 real-time PCR System (Applied Biosystems). The amplification specificity was confirmed by melting curve. The sequences of primers for rat β-actin [31], Collagen I and Aggrecan [32], Collagen II [33], ADAMTS5 (aggrecanase-2) [34], Tissue Inhibitors of Metalloproteinase (TIMP)2 [35], TNFα [36], Bax, Fas, FasL, Caspase2, Caspase3, Caspase8, and Caspase9 [37], and alfa-smooth muscle actin (α-SMA) [38] were all previously described and their efficiency was confirmed by sequencing their conventional PCR products. The primers for rat Matrix Metalloproteinase (MMP)3 (sense: 5′-ACCTATTCCTGGTTGCTG-3′; anti-sense: 5′-GGTCTGTGGAGGACTTGTA-3′), MMP13 (sense: 5′-CTGACCTGG- GATTTCCAAAA-3′; anti-sense: 5′-ACACGTGGTTCCCTGAGAAG-3′), TIMP1 (sense: 5′-CCTCTGGCATCCTCTTGT-3′; anti-sense: 5′-TTGATCTCATAACGC- TGGT-3′), and Proliferating Cell Nuclear Antigen (PCNA) (sense: 5′-CCAGGG- CTCCATCCTGAA-3′; anti-sense: 5′-CCCAGCAGGCCTCATTGAT-3′) were designed with Primer Premier Version 5.0 software and their efficiency was confirmed by sequencing their conventional PCR products.

### Terminal Deoxynucleotidyl Transferase dUTP nick end Labeling (TUNEL) Assay

Apoptosis was examined *in situ* using a TUNEL assay according to the manufacturer’s instructions (Roche, Mannheim, Germany). Briefly, sections were deparaffinized, rehydrated, pretreated with protease K (10 µg/ml, Sigma) for 20 min, and blocked with 3% bovine serum albumin for 20 min at room temperature. The sections were incubated with TUNEL reaction mixture for 1 h at 37°C and covered with fluorescence mounting medium (Zhongshan-Golden-Bridge-Biotechnology, Beijing). Confocal microscopic images were acquired using a Zeiss laser-scanning microscope (LSM 510).

### Immunohistochemical (IHC) Staining

IHC staining was performed with a two-step detection kit (Zhongshan-Golden-Bridge-Biotechnology) as described previously [27]. The primary antibodies were MMP3 (Abcam, 1∶100 dilution), caspase3 (Cell Signaling Technology, 1∶1000 dilution), and α-SMA (Abcam 1∶100 dilution).

### Statistical Analysis

Statistical analysis was performed using SPSS version 11.0 for Windows. All data were presented as mean ± SEM. Following confirmation of normal data distribution, all data between the experimental and control groups were analyzed using Student’s *t* tests with *P* values <0.05 considered to be statistically significant.

## Results

### Confirmation of Injection into Upper Compartment

To confirm the injection site in the upper compartment of the TMJ, one rat was preliminarily dissected after injection of fast green solution into the upper compartment. The needle was inserted right under the root of the zygoma, beneath the temporal fossa into the upper compartment. The green stained region was mainly limited to the upper compartment (Fig. 1B).

### Ultrastructural Changes in Disc

**Figure 2:**
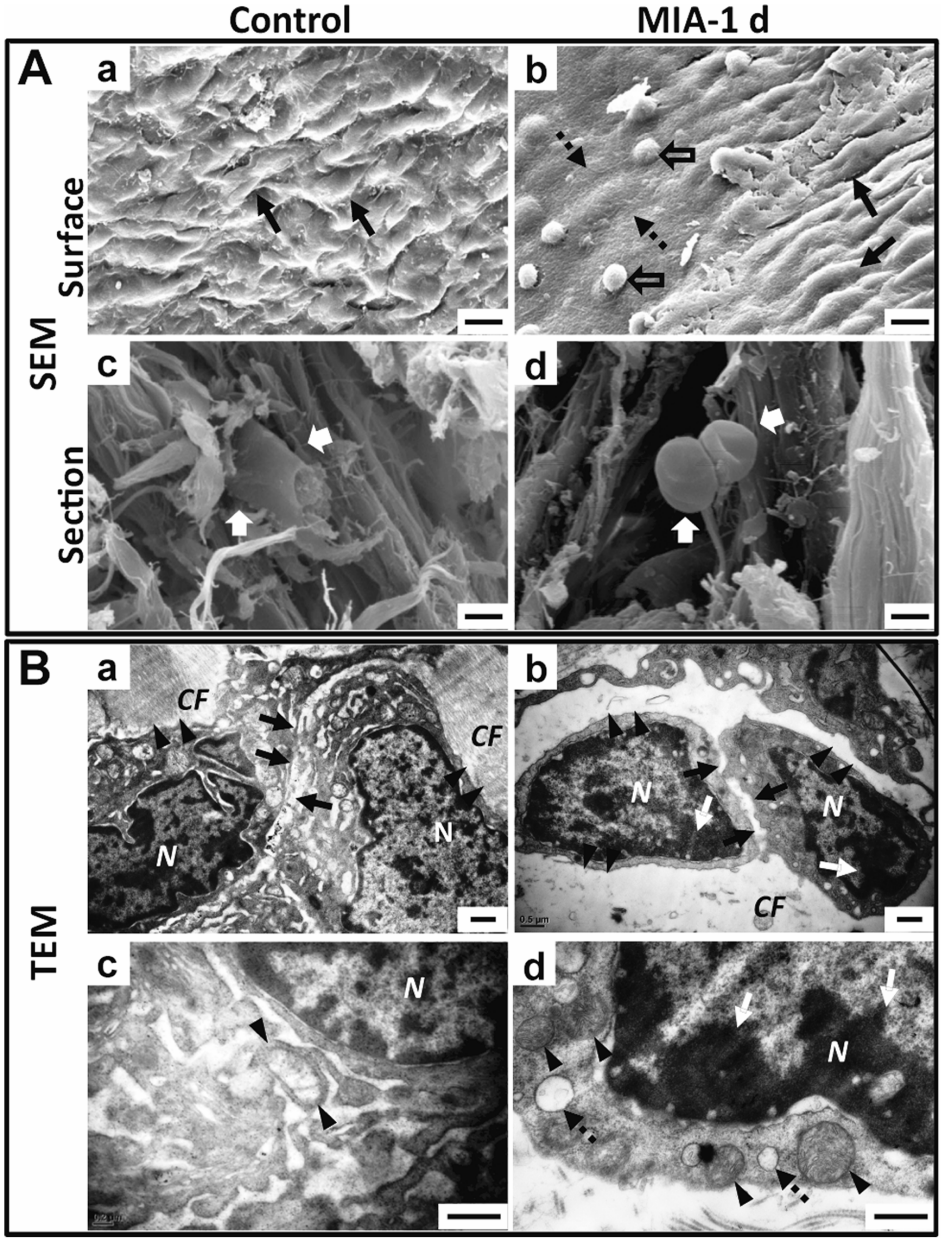
Ultrastructural changes in disc 1 day after MIA injection into upper compartment of TMJ. A. SEM view of the disc. (a). The furrowed surface (arrow) of the control joint. (b). The surface 1 day after MIA treatment, showing regional flattening in the intermediate zone (dotted arrow) with residual gelatin condensed to a mass (hollow arrow), surrounded by an area with a rough and uneven surface (arrow). (c). Section view of the control disc showed the disc cells studded in the collagen fibrils (white arrow). (d). Section view of the disc of the MIA-treated group showed that disc cells were crimpled and detached from the collagen fibrils (white arrow). B. TEM view of the intermediate zone of the disc. (a). Cells in the control disc were closely attached to the collagen fibrils (arrowhead) and cell junctions were tight (arrow). (b). The cells in the MIA-treated disc were shrunken with condensed chromatin (white arrow), detached from the disrupted ECM (arrowhead), and had loose cell junctions (arrow). (c). Mitochondria were regularly tubular-shaped (arrow) around the nuclei in the control disc. (d). Chromatin compaction (white arrow), swollen mitochondria (arrowhead), and vacuolar degeneration (dotted arrow) were observed in the disc cells 1 day after MIA treatment. (CF: collagen fibers; N: nucleus; Bar = 50 µm in A-a, b; Bar = 20 µm in A-c, d; Bar = 0.5 µm in B).

To determine whether MIA injected into the upper compartment of the TMJ could diffuse into the lower compartment, the ultrastructure of the TMJ disc was evaluated by SEM and TEM 1 day after injection of MIA or saline (Fig. 2). SEM showed that the surface of the disc in the control group was furrowed and covered by an evenly distributed gelatinous layer, whereas the intermediate zone of the disc in the MIA group lost these features and presented a limited region with a thinner and smooth surface surrounded by areas with a rough and uneven surface. From the section view of the intermediate zone, disc cells in the control group inserted into the collagen fibrils, whereas the disc cells in the MIA group were crimpled and rounded, detached from the surrounding collagen fibrils (Fig. 2A).

TEM showed that the disc cells of the intermediate zone in the control group were surrounded by a dense, collagenous extracellular matrix (ECM) and the cell junction was tight, with ovoid-shaped mitochondria around the nucleus. However, the disc cells in the MIA group underwent morphological changes, including cell shrinkage, condensation of the cytoplasm and nucleus, cell membrane detachment from the surrounding collagen fibrils, and loosened cell junctions accompanied by the disrupted ECM. Some cells even presented features of apoptosis, such as chromatin compaction, swelling mitochondria, and vacuolar degeneration (Fig. 2B).

### Dose-dependent Histopathologic Changes in TMJ

**Figure 3:**
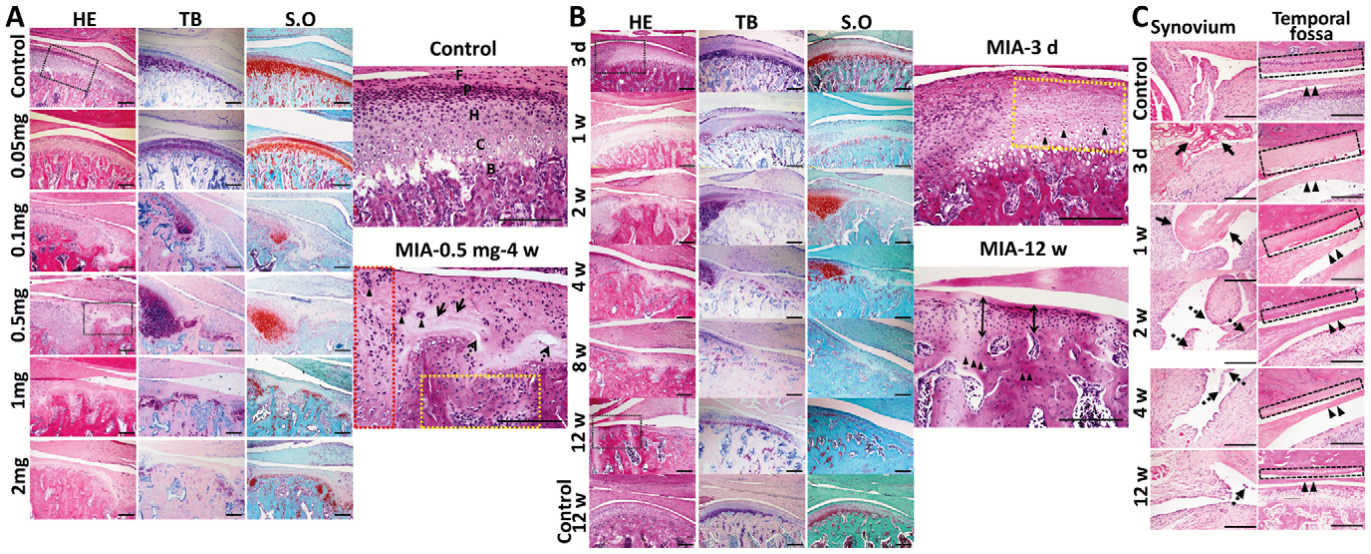
Dose- and time-dependent histopathologic changes in TMJ tissues. TMJ was sectioned in saggital for HE, TB and S.O staining. A. Dose course (0.05 mg, 0.1 mg, 0.5 mg, 1 mg, 2mg per joint) of MIA 4 weeks post-injection. Black frames are magnified. Condylar cartilage in the controls stained purple blue by TB and red by S.O; (F: fibrous layer; P: proliferative layer; H: hypertrophy layer; C: calcified layer; B: subchondral bone.). Typical OA-like lesion induced by 0.5 mg MIA, including regional loss of chondrocytes (arrow), chondrocyte cluster formation (arrowhead), horizontal cleft (dotted arrow), peripheral chondrocyte proliferation (red frame), and subchondral bone erosion with adjacent bone marrow full of fibroblast-like cells (yellow frame). Lesions staining by TB and S.O was uneven. (Bar = 200 µm) B. Time-dependent changes in the condyle following MIA injection (0.5 mg/joint; 3 days to 12 weeks). Black frames were magnified. After three days, chondrocytes in the anterior and central areas of the cartilage were lightly stained with nuclear condensation (arrowhead). At 12 weeks, thin cartilage (double arrow) and sclerotic subchondral bone (arrowhead) replaced the lesion. (Bar = 200 µm) C. Time-dependent changes in the synovium, disc, and temporal fossa following MIA injection. Time-dependent changes in synovitis (fibrin-like exudates: arrow; proliferative villi of the synovium: dotted arrow), hypo-cellular change and thinning of the disc (arrowhead), and destruction of temporal fossa cartilage (black frame) following MIA induction are shown. (Bar = 300 µm).

To understand the effects of MIA on the TMJ, the morphology of the TMJ was examined for 4 weeks after injection with saline or increasing doses of MIA (Fig. 3A). In the control TMJ, HE staining showed that the condylar cartilage was a regular alignment of multilayer chondrocytes. S.O and TB staining showed that the hypertrophic layer was stained red and metachromatically purple, respectively, indicating abundant proteoglycans in the condylar cartilage. In the 0.05 mg MIA group, HE staining showed that the condylar cartilage was slightly decreased in cell number and thickness as compared with the control. S.O and TB staining showed slight decreases in cartilage proteoglycans. In the 0.1 mg MIA group, discontinuousness of the hypertrophic layer with peripheral cartilage thickening was observed. However, in the 0.5 mg group, HE staining showed severe discontinuity of the four-layer cartilage, regional loss of chondrocytes, peripheral proliferation and clustering of chondrocytes, a disorganized matrix network, horizontal clefts, and subchondral bone resorption with adjacent bone marrow filled with fibroblast-like cells. TB and S.O staining showed severe loss of staining in irregularly arranged chondrocytes and enhanced staining at the periphery. In the 1 mg and 2 mg MIA groups, complete loss of chondrocytes, severe thinning of cartilage, and subchondral bone erosion were evident in the lesion, but without peripheral clustering of chondrocytes and thickening of the cartilage. Typical OA-like destruction of the cartilage and erosion of the subchondral bone were observed in the 0.5 mg MIA group [39]. Therefore, 0.5 mg was defined as the minimum effective dose of MIA for induction of typical OA-like lesions in the rat TMJ.

### Time-dependent Histopathologic Changes in TMJ

To further characterize the development of OA, the major structures of the TMJ were evaluated after injection of 0.5 mg MIA at different time points for up to 12 weeks (Fig. 3B, C).

With regard to the condyle, chondrocytes disappeared following MIA induction and the matrix was less stained within the proliferative zone in the anterior and central areas of the condyle corresponding to the load-bearing region. Additionally, scattered cells with nuclear condensation were evident after 3 days. Loss of chondrocytes in all of the cartilage layers with no matrix staining was observed by 1 week. In addition to the above features, regional osteolysis and peripheral chondrocyte proliferation with deep matrix staining were observed by 2 weeks. By 4 weeks, typical OA-like lesions were observed, as described for the 0.5 mg MIA group in the dose course. By 8 weeks, fibrosis in the lesions was evident and the subchondral bone was developing sclerosis. By 12 weeks, the condylar lesions were fully repaired by sclerotic subchondral bone and thin cartilage with disorganized chondrocytes. These changes over the 12-week period were not due to aging effects when compared with the control group. (Fig. 3B).

Time-dependent changes, including synovitis, disc thinning, and the destruction of temporal fossa cartilage following MIA induction, are shown in Fig. 3C. Massive fibrin-like exudates were observed in the upper compartments of TMJs in the experimental group by 3 days to 1 week after MIA injection, but not in the control group. Abundant proliferative villi consisting of multi-layer synovial lining cells and apparent infiltrated mononucleated cells were present in the upper compartment by 2 weeks. The synovial villi decreased and became smaller by 4 weeks and nearly disappeared by 12 weeks. Chondrocytes were almost lost in the cartilage of the temporal fossa and intermediate zone of the disc by 3 days after MIA injection. Until 2 weeks,there were almost no further changes in the disc and temporal fossa. From 4 weeks to 12 weeks, the disc and the cartilage of the temporal fossa became thinner, but the subchondral bone of the temporal fossa remained intact and no disc perforation was observed.

### Radiographic Changes in Subchondral Bone

**Figure 4:**
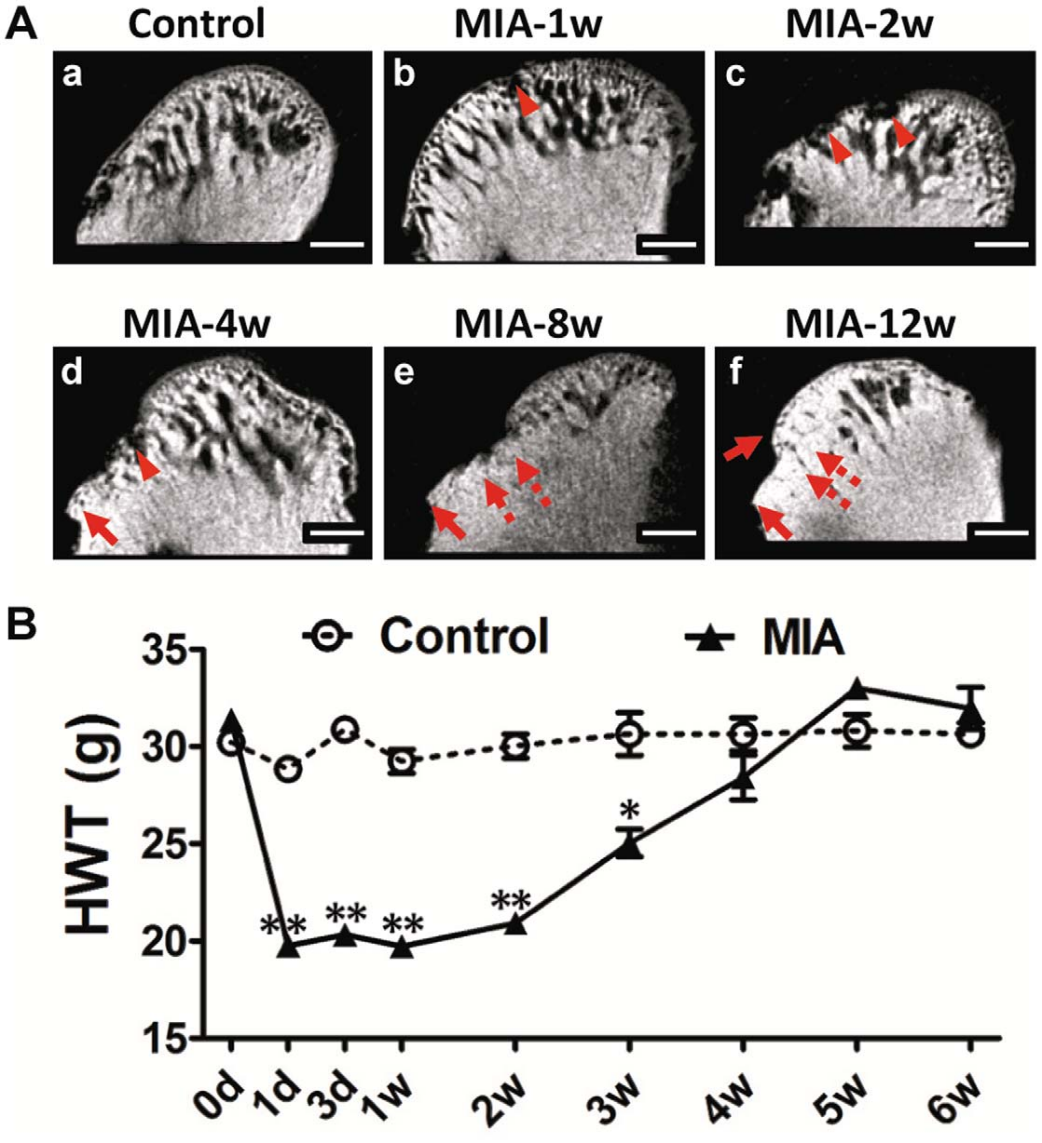
Time-dependent radiographic changes in condylar subchondral bone and nociceptive responses following MIA injection (0.5 mg/joint). A. Representative images of the condyle by MicroCT scanning with a sagittal section view demonstrated. (a). Control condyle showed intact subchondral bone with a smooth, continuous surface. (b). Regional loss of surface bone (arrowhead) occurred in the frontal bevel of the condyle by 1 week. (c). Multiple erosions of subchondral bone (arrowhead) were observed by 2 weeks. (d). Erosion in the subchondral bone grew deeper and was much more extensive with obvious defects (arrowhead) and osteophyte formation (arrow) by 4 weeks. (e and f). Sclerotic changes (dotted arrow) and osteophytes (arrow) were evident by 8 weeks and 12 weeks. (Bar = 300 µm) B. Changes in animal nociceptive response after MIA injection into TMJ. The HWT was significantly decreased in the first 3 weeks after MIA injection, but gradually recovered to control levels from 4 weeks post-injection. All data were presented as mean ± SEM. (n = 3; **P<0.01; *P<0.05).

To fully understand the changes in the subchondral bone after MIA-injection (0.5 mg/joint), radiographic changes in the condyle were evaluated by MicroCT scanning (Fig. 4A). On sagittal images, the bone surface of the control condyle was smooth and continuous, whereas the bone surface of the anterior and central areas of the condyle was discontinuous by 1 week after MIA injection. Multi-erosions, characterized by translucency disrupting the bone surface of the load bearing areas, grew deeper and more extensive with obvious defects from 2 to 4 weeks. By 8 weeks, the bone surrounding the lesion became sclerotic. By 12 weeks, the lesion was replaced with smooth but sclerotic bone. Osteophytes began to present from 4 weeks until 12 weeks after MIA injection.

### Hyperalgesia of TMJ after Induction of TMJOA

To understand the relationship between the nociceptive response and histopathological changes, the HWT was measured at different time points after MIA injection (0.5 mg/joint). (Fig. 4B). The HWT significantly decreased 24 h after MIA injection (*P*<0.01), remained at a decreased level until 3 weeks (*P*<0.05), but then gradually recovered to baseline by 4 weeks, as compared with the control group.

### Induction of Condylar Chondrocyte Apoptosis

**Figure 5:**
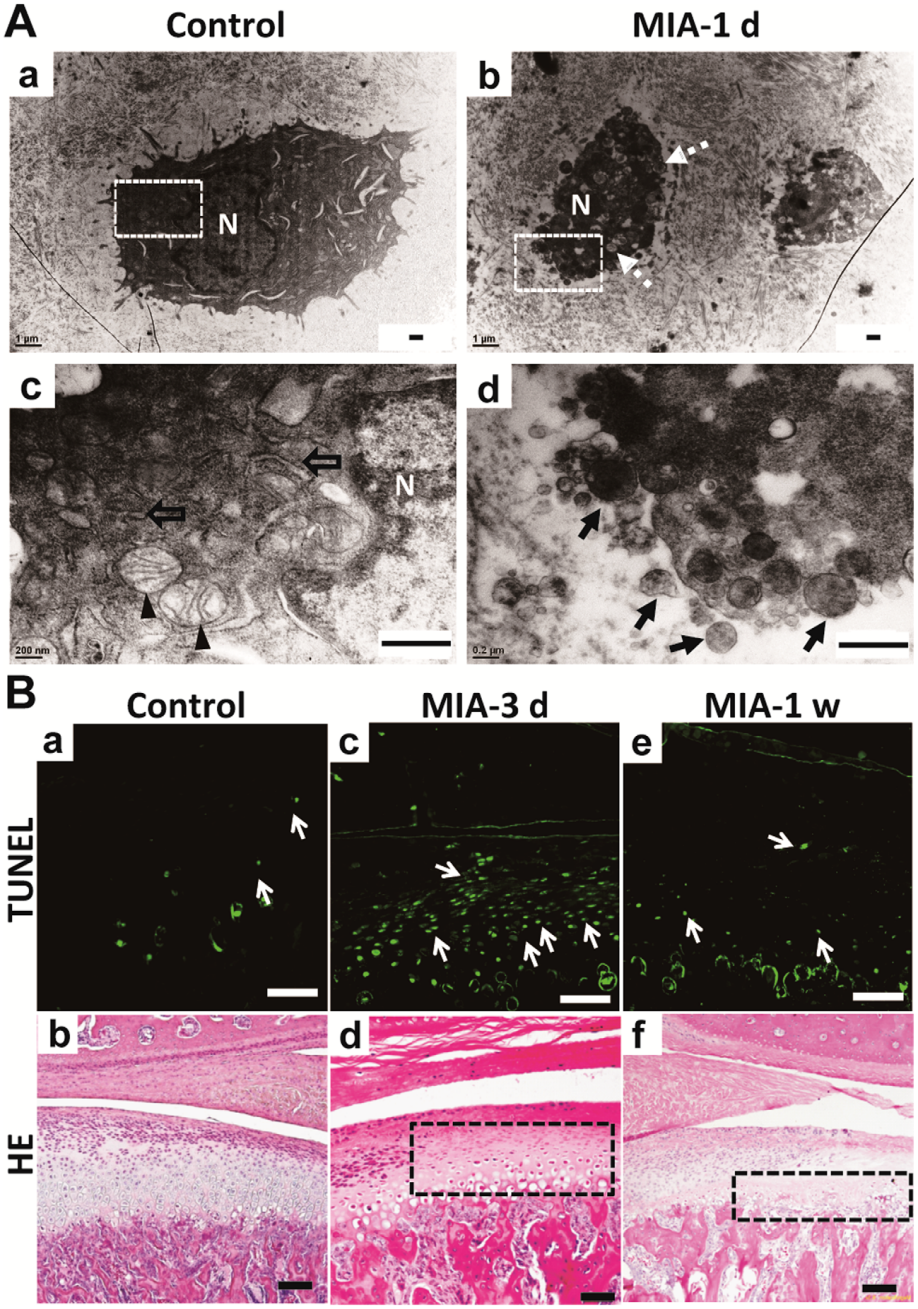
Apoptosis of chondrocytes in condyle after MIA treatment. A. TEM view of condylar chondrocytes. (a). The chondrocytes in the control group were polygonal. (b). Chondrocytes treated by MIA were shrunken with vacuolar degeneration after 1 day (dotted arrow). (c). Magnified photograph of the white frame in (a). Abundant mitochondria (arrowhead) and endoplasmic reticulum (hollow arrow) were observed around the nuclei (N) in the control chondrocyte. (d). Magnified photograph of the white frame in (b). Apoptotic bodies (black arrow) were observed in the chondrocyte following MIA induction. (Bar = 0.5 µm) B. Comparison of TUNEL assay and HE staining results. (a) There were few apoptotic chondrocytes (arrow) in the control group and the corresponding HE staining shown in (b). (c). Diffuse apoptotic chondrocytes were observed in the region corresponding to the lightly stained area with nuclear condensation of HE staining (black frame in d) at 3 days post-MIA injection. The TUNEL positive chondrocytes almost disappeared (e) due to the extreme loss of chondrocytes as shown by HE staining (black frame in f) at 1 week. (Bar = 80 µm).

To understand the mechanism underlying MIA-induced chondrocyte loss in the condylar cartilage, TEM examinations and a TUNEL assay were performed following MIA injection (0.5 mg/joint). TEM showed that the chondrocytes in the control group were polygonal with abundant mitochondria and endoplasmic reticulum, whereas chondrocytes in the group treated with MIA showed typical apoptotic features, including cell shrinkage, nuclear condensation, vacuolar degeneration, and apoptotic bodies, after 1 day (Fig. 5A). Three days after MIA injection, TUNEL-positive chondrocytes were observed diffusely in the area corresponding to the region with HE unstained nuclei, but not in the control group. However, 1 week after MIA injection, the TUNEL positive chondrocytes almost disappeared in the same region due to the extreme loss of chondrocytes as shown by HE staining (Fig. 5B).

### Expression of Metabolism and Apoptosis Related Genes of Condyle after MIA Injection

**Figure 6:**
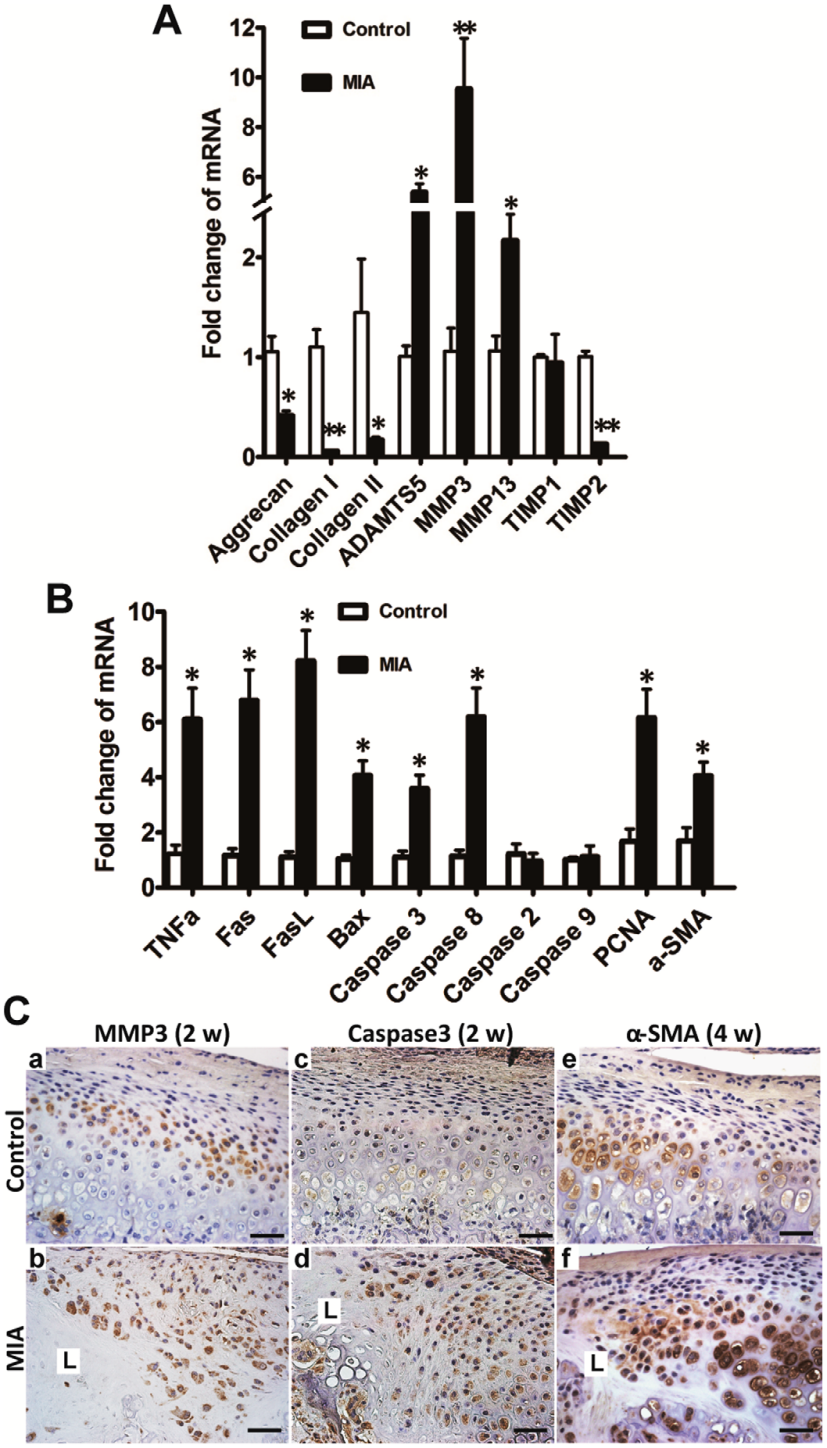
Changes in gene and protein expression in condyle following MIA injection were evaluated by real-time PCR and IHC, respectively. A. Two weeks after MIA injection, anabolism-associated aggrecan and collagen I and II were downregulated compared with the control group. Catabolism-associated MMP3, MMP13, and ADAMTS5 were upregulated and TIMP2, but not TIMP1, was correspondingly downregulated. B. Two weeks after MIA (0.5 mg) injection, apoptosis-associated genes of the death receptor family, such as, TNFα, Fas, FasL, caspase8, caspase3, and BAX, but not caspase2 and caspase9, were significantly elevated in the MIA injection group; PCNA and α-SMA, representing proliferation and fibrous restoration, respectively, were upregulated (mean ± SEM; n = 6; **P<0.01; *P<0.05). C. There were very few chondrocytes left in the lesion labeled as L 2 weeks after MIA (0.5 mg) injection. MMP3 was mainly expressed in the hypertrophic layer in the control cartilage (a). Diffuse staining of MMP3 was observed in the chondrocytes adjacent to the lesion (L) at 2 weeks (b). Caspase3 was rarely expressed in the control cartilage (c). Enhanced staining of caspase3 was observed in the proliferative and hypertrophic layers adjacent to the lesion (L) at 2 weeks (d). Expression of α-SMA was mainly in the hypertrophic chondrocytes in the control group (e). Stronger staining of α-SMA was observed adjacent to the lesion (L) at 4 weeks (f). (Bar = 40 µm).

To further understand the molecular events underlying condylar destruction following MIA induction (0.5 mg/joint), the expressions of genes related to the metabolism of cartilage and bone and apoptosis were examined from the condylar head contain both cartilage and subchondral bone by real-time PCR and IHC 2 weeks after MIA injection. As compared with the control group, mRNA expression of main matrix components, including aggrecan and collagen I and II, were significantly downregulated. However, mRNA expression of the matrix degrading proteases MMP3, MMP13, and ADAMTS5 were significantly upregulated in the MIA group. In contrast, TIMP2, but not TIMP1, was correspondingly downregulated (Fig. 6A). MIA induction resulted in a significant increase in the expression of the proapoptotic genes of the death receptor family, such as Fas, FasL, caspase8, caspase3, and BAX, but not caspase2 and caspase9 (Fig. 6B). PCNA and α-SMA, markers of proliferation and fibrosis [40], [41], respectively, were also upregulated in the MIA group (Fig. 6B). Moreover, IHC showed that MMP3 was mainly expressed in the hypertrophic layer in the control cartilage, but diffuse staining of MMP3 was observed in the chondrocytes adjacent to the lesion. Stronger staining of caspase3 was observed diffusely in the proliferative and hypertrophic layers adjacent to the lesion as compared with the control group. Expression of α-SMA was mainly in the hypertrophic chondrocytes in the control group, whereas it was enhanced in the chondrocytes of the proliferative and hypertrophic layers adjacent to the OA-like lesion by 4 weeks after MIA injection (Fig. 6C).

## Discussion

In this study, we provided multi-level data to show that a comprehensive rat model of TMJOA could be successfully established through MIA injection into the upper compartment of the TMJ. First, electron microscopy showed that the intermediate zone of the disc loosened to facilitate the diffusion of MIA into the lower compartment. Second, histopathologic analysis illustrated that typical OA-like lesions in the TMJ, including degenerative changes in the condyle, disc, and temporal fossa, as well as synovitis, were induced by MIA in a dose- and time-dependent manner. Third, subchondral bone destruction, which is characteristic of the early stage of OA, and sclerosis, which is seen during the later stage of OA, were observed by MicroCT scanning. Fourth, the molecular analysis revealed that chondrocytic apoptosis and the imbalance between the anabolism and catabolism of cartilage and subchondral bone might account for the advanced condylar destruction following MIA induction. Fifth, nociceptive responses increased in the early stages corresponding to the presence of synovitis. To the best of our knowledge, this is the first report to demonstrate that MIA can effectively induce typical OA-like lesions in the TMJ of a rodent species.

The histopathologic features of MIA-induced lesions in the rat TMJ were similar to that of TMJOA. The present study revealed a typical time- and dose-dependent degeneration of TMJ tissues, showing the progress of cartilage degradation, erosion, osteophyte formation, and sclerosis in the subchondral bone, synovitis, and thinning in the disc and temporal surface. The current results are similar to the previous description of TMJOA [2]. The lesions were specifically limited to the load-bearing areas of the condyle. Although MIA was injected into the upper compartment and should have a more direct action on the surface of the temporal fossa than on the condyle, the destruction of condylar cartilage and subchondral bone was more severe than that of the temporal fossa. This feature is similar to the clinical and experimental observations that the condyle is active and undergoes greater destruction and remodeling [42], [43], [44]. Interestingly, the disc did not prevent MIA from penetrating into the lower compartment. In as little as 24 h the disc cells underwent apoptotic changes, such as cell body shrinkage and mitochondrial breakage, accompanied by disruptive ECM and loosened junctions between cells and between cells and the ECM. These changes facilitated the penetration of MIA through the disc to the lower compartment after injection into the upper compartment.

The radiographic findings of MIA-induced lesions in the rat TMJ were similar to that of TMJOA. The typical clinical radiographic findings for the condyle are erosion, sclerosis, and osteophytes [3]. All of these features could also be observed with MicroCT in our MIA-induced rat TMJOA model. Moreover, the radiographic features of our TMJOA model corresponded well to the histopathologic changes. Therefore, this MIA model provides detailed histopathologic changes for the corresponding radiographic changes. In addition, this model can also be used for in vivo radiographic analysis of subchondral bone to understand the pathogenesis of TMJOA, as it already known for knee OA [17].

Nociceptive responses of MIA-induced TMJOA corresponded to the observed histopathologic changes. Pain is one of the predominant clinical features of OA and it may arise from the soft tissues around the joint or the subchondral bone undergoing destruction [45]. Therefore, a successful animal model of OA should have appropriate nociceptive responses corresponding to its histopathologic changes. The HWT is usually used evaluating TMJ nociceptive responses and is inversely associated with TMJ inflammation and pain [26]. We observed that TMJ hyperalgesia corresponded to the observed histological and radiographic changes in the MIA-induced TMJOA. Specifically, the hyperalgesia of TMJ in the first week after MIA injection could be mainly inflammatory response, whereas in the 2–4 weeks after MIA injection, the hyperalgesia could well correspond to the subsequent pronounced destruction of condylar cartilage and subchondral bone erosion. When the synovitis was alleviated and cartilage damage was repaired by fibrous tissue and the subchondral bone underwent a sclerotic change, the nociceptive responses correspondingly returned to baseline. This was consistent with known clinical features. For example, patients often experience severe pain during the active destructive phase of TMJOA with synovitis [46] and feel alleviation over time [47], [48]. However, the hyperalgesia in our TMJOA model recovered to the control level within 6 weeks, whereas last-long hyperalgesia was observed in the MIA-induced knee joint OA model [49]. Although the reasons for this difference are unknown, it might be related to the difference in the degree of cartilage damage induced by MIA in different joints, since the same dose of MIA induces more severe cartilage loss in the knee joint than in the TMJ [49]. It might also be related to the properties of the different types of cartilage, i.e., the TMJ is covered with fibrocartilage and the knee joint with hyaline cartilage. Since the hyperalgesia of the TMJ correspondingly reflected the degree of lesions induced by MIA, our results also suggested that MIA-induced TMJOA can be used for evaluating osteoarthritic pain in the TMJ.

MIA induced TMJOA through chondrocyte apoptosis and the disturbance of cartilage and subchondral bone metabolism. MIA could sensitively induce chondrocyte apoptosis as early as 1 day after MIA injection and condylar apoptosis reached a peak on day 3, leading to hypocellular changes in the cartilage and disc. Chondrocyte apoptosis in the early stages could be an important initiator of cartilage degeneration. Genes of the death receptor family, such as Fas and FasL, have been reported to be related to chondrocyte apoptosis [50], [51], [52]. Gene expression of the death receptor family and IHC staining of caspase3 further showed that the apoptotic process appeared to be caspase-dependent. This is consistent with previous studies of OA in the knee [18], [22] and discectomy-induced TMJOA [53]. In addition, cartilage degeneration also results from the imbalance between anabolism and catabolism due to increased matrix degrading proteases and decreased synthesis of matrix [54]. Although the genes expression was evaluated from the condylar head containing both cartilage and subchondral bone, the results showed that the catabolic genes MMP3, MMP13, and ADAMTS5 were elevated in the condylar head, whereas the anabolic genes aggrecan and collagen I and II were decreased in the condylar head. The observed changes in gene expression were similar to previous reports of experimental OA or clinical OA [55], [56]. Therefore, MIA-induced imbalances in gene expression with regard to cartilage metabolism and subchondral bone could also be an important factor contributing to condylar deterioration.

The present model of TMJOA has advantages and disadvantages. Lack of severe histopathologic changes associated with TMJOA, such as vertical splitting in the cartilage, exposure of subchondral bone, and disc perforation, could be one of the disadvantages for MIA-induced TMJOA model. In contrast, advantages include the signs of reconstruction, including hypertrophic reactions in the cartilage surrounding lesions, fibrous restoration as represented by α-SMA [41] overexpression in the proliferative cells, and hypertrophy of the chondrocyte layer at 4 weeks post-MIA injection. In addition, sclerosis of subchondral bone and osteophyte formation were observed in the later stages, which mimics the typical clinical features [42]. Lesion repair following MIA injection was also reported in a rabbit model [13], [23]. TMJOA is a self-limiting disease and reconstruction plays an important role [2]. However, reconstruction is rarely seen in TMJOA models induced by methods other than MIA. Although several animal models of TMJOA have been established, our multi-level data suggest that the present rat model accurately mimicked most of the clinical features of TMJOA.

In conclusion, the present study demonstrated a reliable and simple rat model of TMJOA induced by intra-articular injection of MIA into the upper compartment. The histopathologic, radiographic, behavioral, and molecular changes of this model will help us to understand the progression of TMJOA and to facilitate future TMJOA-associated researches.
